# A Patient with Severe Leptospirosis Treated with Cytokine Removal and High-Dose Corticosteroids

**DOI:** 10.3390/idr14050068

**Published:** 2022-08-27

**Authors:** Jure Fluher, Iva Cestar, Katja Jerenec, Žiga Kalamar, Zvonko Baklan, Andrej Markota

**Affiliations:** 1 Medical Intensive Care Unit, University Medical Centre Maribor, Ljubljanska ulica 5, 2000 Maribor, Slovenia; 2 Medical Faculty, University of Maribor, Slomškov Trg 15, 2000 Maribor, Slovenia; 3 Department of Infectious Disease and Febrile Conditions, University Medical Centre Maribor, Ljubljanska ulica 5, 2000 Maribor, Slovenia

**Keywords:** leptospirosis, circulatory failure, inflammatory cytokine response, corticosteroid therapy, acute heart failure, case report

## Abstract

Leptospirosis is an ubiquitous zoonosis with significant morbidity and mortality. Approximately 10 percent of human infections evolve into a severe form, with a sepsis-like disease, multiorgan failure, and significant mortality rate. The cornerstone of treatment of severe disease is antibiotic therapy, with the aims of preventing complications, reducing the duration of disease, and ultimately reducing mortality. The initiation of antibiotic chemotherapy can precipitate a febrile inflammatory reaction, also known as a Jarisch–Herxheimer reaction. We present a case report of a patient with severe leptospirosis, complicated by multiorgan failure with severe circulatory failure of distributive and cardiogenic etiology, possibly as a consequence of the Jarisch–Herxheimer reaction. The patient was treated with antimicrobial therapy and other supportive measures along with high-dose corticosteroid therapy, long-term mechanical ventilation, high-dose vasopressor therapy, and continuous veno-venous hemodiafiltration with extracorporeal cytokine removal.With this case, we would like to report on a patient presenting with two neglected diseases in our part of Europe, who was treated with novel therapeutic strategies.

## 1. Introduction

Leptospirosis is an ubiquitously distributed zoonosis caused by pathogenic spirochetes of Leptospira species. Human infection results after exposure to environmental sources, such as contaminated water or soil, or infected animal sources, such as tissue or urine. The disease spectrum ranges from subclinical infection to a life-threatening disease associated with multiorgan failure. The clinical presentation of leptospirosis is biphasic, consisting of the acute or septicemic phase lasting from two to nine days, followed by a period of apparent improvement, and the immune phase with potential multiorgan failure driven by antibody and cytokine production. The mortality rate of severe leptospirosis ranges between 5 and 15% [1,2].

The main contributor to the severity of leptospirosis disease is the cytokine storm that potentially arises in the second (immune) phase of the disease. Multiorgan failure associated with leptospirosis may comprise of acute renal failure, thrombocytopenia, hepatic involvement, acute respiratory failure, and circulatory failure. Acute renal failure occurs with a prevalence of 16 to 40% of cases of severe disease, is usually nonoliguric, and may require renal replacement therapy, although renal recovery is usually complete. Thrombocytopenia occurs in more than one-third of cases, is transient, and is not associated with disseminated intravascular coagulation. Hepatic involvement with jaundice, which is also known as Weil’s disease, also occurs frequently, but does usually not lead to liver failure. Pulmonary involvement ranges from cough and dyspnea to severe intra-alveolar hemorrhage and ARDS, which is associated with poor outcomes. Cardiac involvement is reported in the form of EKG repolarization abnormalities, arrhythmias, pericarditis, and, in severe cases, myocarditis with circulatory failure [1,2,3,4]. 

The cornerstone of treatment for severe leptospirosis, besides supportive therapy, is antibiotic therapy with penicillin, ceftriaxone, doxycycline, or cefotaxime, which prevents the development of the immune phase or significantly reduces its duration. The initiation of chemotherapy in spirochetal disease may precipitate a febrile Jarisch–Herxheimer reaction (JHR). This is an acute inflammatory reaction due to exposure to bacterial endotoxins and microbial antigens that are released by microorganism destruction. It is a phenomenon most often associated with spirochetes, including syphilis, Lyme disease, relapsing fever, and leptospirosis. The incidence of JHR in leptospirosis patients is approximately 9% [5,6,7].

## 2. Case Presentation

### 2.1. Emergency Department (ED)

A 51-year-old previously healthy male worker in forestry presented to the emergency department (ED) with a 4-day history of fever (up to 39 °C), headache, myalgias, diarrhea, and oligo-anuria. His blood pressure on arrival to ED was 90/60 mmHg with a heart rate of 125/min; he was febrile (39 °C), and his oxygen saturation was 98% on ambient air. Initial laboratory investigations revealed an elevated serum lactate (5.1 mmol/L—approximately three times the upper limit), elevated leukocytes (12 × 10^9^/L) with a left shift and normal platelets (153 × 10^9^/L), elevated C-reactive protein (474 mg/L—app. 100 times the upper limit) and procalcitonin (49 mcg/L—app. 100 times the upper limit), and elevated creatinine (468 µmol/L—app. 4.5 times the upper limit) and urea (17.1 mmol/L—app. 2.5 times the upper limit). Point-of-care ultrasound (POCUS) revealed a normal functioning left ventricle with an ejection fraction (EFLV) of 60% and more than 50% respiratory variability in the diameter of the inferior vena cava. After samples for microbiological analysis were obtained, antibiotic therapy with ceftriaxone was started. Hypotension persisted despite fluid resuscitation (2000 mL of balanced crystalloids during the first two hours) and an infusion of noradrenaline was required to maintain his blood pressure. 

### 2.2. Intensive Care Unit (ICU) 

He was admitted to ICU 4 h after arrival to ED; in the 4 h following admission, hemodynamic instability worsened. Noradrenaline at 1.55 mcg/kg/min and argipressin at 0.065 IE/min were required. He was intubated due to the development of respiratory failure and delirium. Additional laboratory tests revealed elevated levels of interleukin-6 (IL-6; 1879 pg/mL), serum lactate increased to 11.9 mmol/L, and platelets decreased to 95 × 10^9^/L. Due to the requirement for high-dose vasopressors and elevated concentrations of IL-6, cytokine removal therapy was initiated 4 h after arrival to ICU. Bedside echocardiography was performed multiple times during the first hours of ICU treatment and subsequently severe systolic dysfunction developed with LVEF decreasing to 25% and left ventricular outflow tract volume–time integral (LVOT VTI) measured at 9 cm. High-sensitivity troponin I increased to 36,807 ng/L at 24 h after admission and nonspecific changes were present on the EKG. In the first 18 h of cytokine removal, vasopressor requirements gradually decreased to noradrenaline and argipressin infusion rates of 0.85 mcg/kg/min and 0.027 IE/min, respectively. Serum lactate gradually decreased to 5.0 mmol/L. No further worsening of systolic function was observed. At 24 h after admission, a positive urine and serum polymerase chain reaction for *Leptospira* species was received. In addition to antibiotic therapy (ceftriaxone 2 g daily), vasopressors, mechanical ventilation, and cytokine removal treatment with high doses of steroids (methylprednisolone 1500 mg daily for three days) were initiated. Because of anuric acute renal failure, continuous veno-venous hemodiafiltration was added to the extracorporeal circuit. Cytokine removal was performed for 72 h. During this time, three membranes were exchanged. IL-6 concentration decreased to 379 pg/mL, lactate decreased to below 2 mmol/L, the noradrenaline infusion rate decreased to 0.2 mcg/kg/min, argipressin could be discontinued, and EFLV increased to 50%. Platelets decreased to 33 × 10^9^/L on day 3 and then gradually increased to around 300 × 10^9^/L over the next 4 days. 

### 2.3. Case Outcome

Antibiotic therapy with ceftriaxone was continued until day 12. Renal replacement therapy was required until day 18. During the ICU stay, ventilator-associated pneumonia developed caused by *Enterococcus faecalis* and *Enterobacter cloacae*; this was successfully treated with piperacillin/tazobactam. The patient was successfully weaned off mechanical ventilation and extubated on day 19. Corticosteroid therapy was gradually tapered to withdrawal on day 32 of ICU treatment. In addition to echocardiography, EKG, and biomarkers, cardiac CT (on day 20) and MRI (two weeks after hospital discharge) were performed in an attempt to explain severe systolic dysfunction. Cardiac CT revealed hemodynamically irrelevant pericardial effusion (maximal diameter of 16 mm) and diffuse myocardial calcinations. Cardiac MRI revealed diffuse signal amplification consistent with septic cardiomyopathy. The patient was discharged to a hospital ward on day 35 and home on day 52.

## 3. Discussion

Our patient presented to the ED with nonspecific flu-like symptoms. After laboratory investigations were obtained, sepsis with acute renal failure was diagnosed. Among the differential diagnoses are acute viral illnesses, such as influenza or Hantaan virus infection, which can cause a pulmo-renal syndrome similar to leptospirosis. In our case, a Hantaan virus infection was excluded after negative serology was obtained. One consideration in our region is also tick-borne diseases, such as ehrlichiosis and anaplasmosis, which can present with thrombocytopenia and, in the case of anaplasmosis, also with pneumonia. As our patient developed respiratory failure and thrombocytopenia in the first 24 h of treatment, the addition of the antibiotic doxycycline could be considered in the absence of other microbiological results.

Although a degree of hemodynamic instability in our patient was present at ED admission, progressive deterioration with immunologic dysregulation (high IL-6), manifested as distributive and cardiogenic shock (elevated troponin I levels, cardiac dysfunction on POCUS, and an increasing requirement for vasopressors) ensued in the 6–10 h after antibiotic administration. The determinants of leptospirosis disease severity are pathogen-related factors, such as infection with a specific Leptospira serogroup and the inoculum size, and host-associated factors, with their immunological profile and associated immune response possibly complicated with cytokine production [2]. 

An etiologic component of JHR to circulatory failure was suspected. However, the incidence of this immunological phenomenon in leptospirosis is not well documented. Diagnosis presents a clinical challenge as it is hard to distinguish from, for example, an allergic reaction to drugs, concomitant viral infection, or possible progression of underlying sepsis [5]. Infection with a specific serogroup and short delay between symptom onset and antibiotic administration are reported risk factors for JHR development. Therapeutic strategies are focused on supportive measures; hemoadsorption with cytokine removal is, according to our knowledge, not reported [5,8].

**Figure 1:**
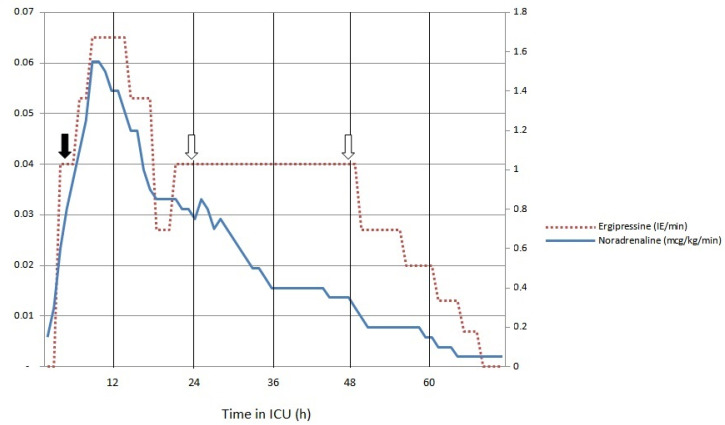
Vaspressor doses in the first 72 h of ICU treatment (solid arrow represents the start of cytokine removal and empty arrows represent the timepoints of hemadsorption membrane changes).

An inflammatory response with destructive immune mechanisms, which evidently developed in our case, falls within the rationale for extracorporeal cytokine removal. The initiation of cytokine removal therapy in our case resulted in the gradual reduction of vasopressor doses (Figure 1).

A possible benefit of corticosteroid therapy in the setting of severe leptospirosis, especially in cases with pulmonary involvement (diffuse alveolar hemorrhage or ARDS), is reported and is based on the nonspecific immunosuppression of high-dose corticosteroids [9]. In our case, a 3-day 1.5 g daily methylprednisolone therapy, with gradual dose tapering, was administered 24 h after ICU admission.

Plasma exchange therapy is also reported as a treatment consideration in severe disease, especially with lung involvement in the form of diffuse alveolar hemorrhage [10].

Cardiac involvement has been described in patients with leptospirosis and patients with suspected JHR of other spirochetal origin, although the prevalence and form is poorly understood. Endocardial inflammation with vasculitic changes has been reported as one of the pathogenic mechanisms of disease in a report of the pathological examination of hearts from patients who died from leptospirosis. Due to associated multiorgan involvement, the cardiac manifestations may possibly be underdiagnosed [11,12].

In our case, we observed circulatory failure with evidence of severe cardiac dysfunction on POCUS, with elevated cardiac biomarkers and nonspecific electrocardiographic repolarization changes. The improvement in cardiac function (improved EFLV and LVOT VTI), normalization of cardiac biomarker levels, and gradual EKG normalization occurred simultaneously with a decrease of inflammatory parameters and hemodynamic stabilization during the 72 h of cytokine removal therapy.

**Figure 2:**
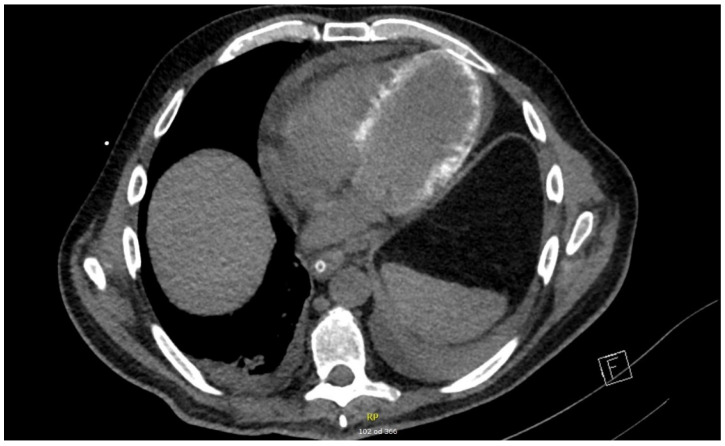
Cardiac CT scan with diffuse myocardial calcinations and pericardial effusion, performed on day 20 of ICU treatment (axial image).

The results of additional investigations that were performed during the resolution phase of disease showed signs consistent with a septic cardiomiopathy. On cardiac CT performed on day 20 in the ICU, a hemodynamically unremarkable pericardial effusion (maximal diameter of 16 mm) and diffuse myocardial calcinations were observed (Figure 2). 

On cardiac MRI, which was performed 6 weeks after full-blown disease manifestation, diffuse subepicardial signal amplification and transmural signal amplification in the anterior, anterolateral, and anteroseptal walls of the left ventricle were present.

## 4. Conclusions

We present a case of a male patient with severe leptospirosis and a possible Jarisch–Herxheimer reaction after antibiotic therapy with ceftriaxone. The ensuing immunological response resulted in multiorgan dysfunction with circulatory failure and a septic cardiomyopathy. A high-dose corticosteroid pulse therapy was administered and cytokine removal was performed for 72 h, during which improvement was observed. 

In patients with suspected spirochetal infection that present with multiorgan failure and circulatory collapse, especially after antibiotic administration, early treatment with hemadsorption and cytokine removal may be considered, and high-dose corticosteroid therapy may be considered after leptospirosis infection is confirmed.
